# Determination of Chromium in Natural Water by Adsorptive Stripping Voltammetry Using *In Situ* Bismuth Film Electrode

**DOI:** 10.1155/2020/1347836

**Published:** 2020-05-14

**Authors:** Nguyen Thị Hue, Nguyen Van Hop, Hoang Thai Long, Nguyen Hai Phong, Tran Ha Uyen, Le Quoc Hung, Nguyen Nhi Phuong

**Affiliations:** ^1^University of Sciences, Hue University, Hue 530000, Vietnam; ^2^Institute of Chemistry, Vietnam Academy of Science and Technology, Ha Noi 100000, Vietnam; ^3^Pham Van Dong University, Quảng Ngãi 570000, Vietnam

## Abstract

Development of adsorptive stripping voltammetry (AdSV) combined with *in situ* prepared bismuth film electrode (*in situ* BiFE) on glassy carbon disk surface using diethylenetriamine pentaacetic acid (DTPA) as a complexing agent and NO_3_^−^ as a catalyst to determine the trace amount of chromium (VI) is demonstrated. According to this method, in the preconcentration step at *E*_dep_ = −800 mV, the bismuth film is coated on the surface of glassy carbon electrodes simultaneously with the adsorption of complexes Cr(III)-DTPA. In addition to the influencing factors, the stripping voltammetry performance factors such as deposition potential, deposition time, equilibration time, cleaning potential, cleaning time, and technical parameters of differential pulse and square wave voltammetries have been investigated, and the influence of Cr(III), Co(II), Ni(II), Ca(II), Fe(III), SO_4_^2−^, Cl^−^, and Triton X has also been investigated. This method gained good repeatability with RSD <4% (*n* = 9) for the differential pulse adsorptive stripping voltammetry (DP-AdSV) and RSD < 3% (*n* = 7) for the square wave adsorptive stripping voltammetry (SqW-AdSV), and low limit of detection: LOD = 12.10^−9^ M ≈ 0.6 ppb (at a deposition potential (*E*_dep_) of −800 mV and the deposition time (*t*_dep_) of 50 s) and LOD = 2.10^−9^ M ≈ 0.1 ppb (at *E*_dep_ = −800 mV and *t*_dep_ = 160 s) for the DP-AdSV and SqW-AdSV, respectively. This method has been successfully applied to analyze chromium in natural water.

## 1. Introduction

In the natural environment, chromium exists in two thermodynamically stable states, Cr(VI) and Cr(III). Its toxicity depends on the level of oxidation; Cr(VI) is the causative agent of cancer through the airways and toxic to humans and other mammals, while Cr(III) at trace levels provides the body with essential minerals. The sources of chromium emissions into the environment are electroplating, metallurgical, dye processing, tanning, minerals, and refractory materials [1, 2]. The form of chromium does not only determine its ecological impact but also determines its movement and variation in the environment. Studies related to the transformation of Cr(VI), the distribution of Cr(III) between inorganic and organic compounds, and the treatment of chromium-contaminated environment are increasingly being considered [3, 4].

There are several sensitive methods for determining chromium such as atomic absorption spectrometry [5, 6], plasma emission spectrometry [7, 8], and capillary gas chromatography [9]. These methods, while highly sensitive, only reach the ppb size detection limit and often require preenrichment and extraction stages, which can lead to sample contamination, complicate processes, and increase the cost of analysis, and therefore, it will be difficult to require frequent and mass analysis of samples in the environment. To overcome these difficulties, in recent years, analysts have successfully applied the AdSV method. This method allows direct determination (i.e., no need to extract or evaporate the sample) of the amount of traces, even super chromium traces in different objects, and achieves very low LOD, size  < 10^−9^–10^−12^ M [10–20].

Another problem raised was the working electrode used in the AdSV method. Currently, most studies on the AdSV method use hanging mercury drop electrodes (HMDEs) [21–34], or static mercury drop electrodes (SMDE) [19, 35]which are expensive and very difficult to fabricate. Research on the use of mercury film electrode (MFE) and bismuth film electrode on glassy carbon disk surface (BiFE), which are less expensive and easier to fabricate, and environmentally friendly BiFE has been improved.

AdSV has been applied in combination with different types of electrodes and stripping voltammetry signaling techniques, and many authors have been successful in determining the amount of traces and super chromium traces in complex objects. Most studies use HMDE electrodes, some use MFE electrodes, and there are also many *ex situ* BiFE electrodes [10, 11, 14, 16, 17, 36] and gold film electrodes (AuFE) [13, 15, 37–39], and the later modified electrodes [20, 36, 40–44] are applied more for chromium analysis in complex objects. The complex ligands used are DTPA [1, 21, 22, 25, 26, 28, 32, 35, 45], triethylenetetramine hexaacetic acid (TTHA) [19, 29, 31, 37], diphenylcarbazide (DPCB) [23, 29], pyrocatechol violet [24, 30], pyrogallol [34], rubeanic acid [33], neo TT [40], and quercetin [20], and the common base ingredients are CH_3_COONa, acetate buffer (CH_3_COOH/CH_3_COONa), and CH_3_COONa/NaNO_3_; all studies analyzed chromium in river water, seawater, groundwater, tea water, and wastewater, and almost no works have analyzed chromium in sediments by the adsorptive stripping voltammetry method and no author has used *in situ* BiFE to analyze chromium in environmental objects. Overall, studies have achieved very low detection limits from 10^−9^ to 10^−10^ M (or from 0.05 ppb to 0.005 ppb).  Table 1 shows the summary of published studies on chromium determination by the AdSV method.

When analyzing Cr(VI) by the AdSV method, Yong et al. [10] and Lin et al. [11] proposed the adsorption mechanism as follows.

The first stage of the adsorptive stripping voltammetry method is the movement of Cr(VI) in the solution to the electrode surface and is reduced by the following reaction.

Cr(VI) + 3e ⟶ Cr(III); *E*_1/2_ = −0.2 V in comparison with 3 M Ag/AgCl/KCl electrode; in the presence of DTPA ions on the interface between the electrode membrane and the solution, Cr(III) ions form complexes quickly and adsorbed onto the electrode surface. The main nature of this complex is unknown, and some authors [21, 46] said that this complex exists mainly as Cr(III)-DTPA^2−^ and a little in Cr(III)-HDTPA^−^, or Khan [47] suggested that the complex of Cr(III) with DTPA exists as [Cr(III) (H_2_O)HY]^−^, [Cr(III)-DTPA]^2−^, and so on.

After complex adsorption, potential scan was conducted from −0.9 V to −1.35 V [10], or from −0.8 V to −1.4 V [11]; as a result of this process, the Cr(III)-DTPA complex is reduced to the Cr(II)-DTPA complex [10, 11]:

(1) $$\begin{array}{c} \text{Cr}\left(\text{III}\right)-\text{DTPA}+\text{e}⟶\text{Cr}\left(\text{II}\right)-\text{DTPA} \end{array}$$

The signal recorded with this reduction is at *E*_1/2_ = −1.15 V [10, 11] and at *E*_1/2_ = −1.1 V in comparison with 3 M Ag/AgCl/KCl electrode.

The role of NO_3_^−^ ions is to oxidize Cr(II)-DTPA complexes into Cr(III)-DTPA complexes.

Thanks to the oxidation of NO_3_^−^, the peak is higher and the sensitivity of the method is increased.

(2) $$\begin{array}{c} \text{Cr}\left(\text{II}\right)-\text{DTPA}\overset{NO_{3}^{-}}{⟶}\text{Cr}\left(\text{III}\right)-\text{DTPA} \end{array}$$

In order to contribute to the development of the AdSV method, we have conducted several studies to determine chromium by the AdSV method using mercury film electrodes, *ex situ* bismuth film electrode, and *in situ* bismuth film electrode. In this paper, we present the results of chromium determination by the AdSV method using *in situ* bismuth film electrode (*in situ* BiFE) in the presence of DTPA as a complexing agent and ion NO_3_^−^ as a catalyst. Literature survey revealed that *in situ* BiFE has never been used for chromium determination by the adsorptive stripping voltammetry.

## 2. Experimental

### 2.1. Apparatus and Reagents

Square wave stripping voltammetric measurements were conducted on Electrochemical Analyzer 797 VA Computrace (Metrohm, Switzerland) accompanied with three electrodes. These electrodes were inserted into the 80 ml capacity electrochemical cell. The working electrode was a glassy carbon rotating disk electrode with *d* = 2.8 ± 0.1 mm; the reference electrode was a 3 M Ag/AgCl/ KCl electrode and the auxiliary electrode was a platinum wire. All measurements were carried out at 25 ± 1°C.

DTPA (diethylenetriamine pentaacetic acid) is used as a complexing agent for chromium. 50.10^−3^ M DTPA solution was prepared by dissolving 4.916 g of DTPA in double-distilled water and then adding 25% aqueous ammonia solution until the pH reaches 6.0.

0.2 M Cr(VI) stock solution was prepared by weighing 7.35 g of K_2_Cr_2_O_7_ (Merck, purity of 95–98%), dissolving, and making it up to 250 ml with double-distilled water. Cr(VI) working solutions such as Cr(VI) 10^−6^ M and Cr(VI) 10^−7^ M are diluted daily from this stock solution.

0.48.10^−3^ M Bi(III) working solution was prepared from 4.8 .10^−3^ M Bi(III) (the type used for analyzing atomic absorption spectrometry from Merck).

Acetate buffer (pH = 6) was prepared from NaCH_3_COO (Merck, purity of 95–98%) and CH_3_COOH (Merck, purity 97%). 2.5 M NaNO_3_ solution was prepared from NaNO_3_ (Merck, purity of 95–98%).

Other metal ionic solutions such as Co(II), Ni(II), Zn(II), Cr(III), Fe(III), and Ca(II) are from the corresponding 1000 mg/l stock solution (the type used for analyzing atomic absorption spectrometry from Merck). Triton X-100 working solution was prepared from Triton X-100 (Merck).

### 2.2. The Working Electrode and Adsorptive Stripping Voltammetric Procedure

In this method, the working electrode is bismuth film electrode created in *in situ* on glassy carbon rotating disk (*in situ* BiFE) and it is formed during the deposition process in the following way: The glassy carbon disk electrode was inserted into the electrochemical cell containing the reference electrode, platinum auxiliary electrode, and analysis solution (0.4.10^−3^ M DTPA, 28.8. 10^−5^ M Bi(III), 5.0.10^−6^ M KBr, 0.4 M NaNO_3_, 0.4 M acetate buffer solution, and Cr(VI)). The glassy carbon electrode was rotated with constant speed and deposition at −800 mV was observed (deposition voltage, *E*_dep_) at a definite time (deposition time, *t*_dep_); in the process, Bi(III) is reduced to Bi^0^ which adheres to the surface of glassy carbon plate forming *in situ* BiFE; at the same time, Cr(VI) is reduced to Cr(III), and then new Cr(III) forms complexes with DTPA in the solution layer close to the electrode surface and Cr(III)-DTPA complex adsorbed onto the surface of *in situ* BiFE, so chromium is enriched on the surface of the *in situ* BiFE [10]. At the end of this period, the electrode stops rotating for 30–60 seconds (equilibration time, *t*_equal_). Subsequently, the potential scan was carried out in a negative potential direction from −800 mV to −1450 mV, and at the same time, the stripping voltammogram was recorded using a certain stripping voltammetry technique, differential pulse adsorptive stripping voltammetry (DP-AdSV), or square wave adsorptive stripping voltammetry (SqW-AdSV). During this period, Cr(III) in the Cr(III)-DTPA complexes is reduced to Cr(II) forming Cr(II)-DTPA complexes and generating the stripping peak current of chromium (*I*_p_) [10]. If NO_3_^−^ is not present in solution, *I*_p_ will be very small, NO_3_^−^ present in the solution will oxidize Cr(II)-DTPA to Cr(III)-DTPA and then Cr(III)-DTPA is electrochemically reduced to Cr(II)-DTPA, and the repeated cycle increases the height of *I*_p_ [10]. In other words, the NO_3_^−^ ion acts as a catalyst. After dissolving, the electrode was cleaned by electrolysis at +400 mV for 30 seconds to dissolve Bi^o^ and other metals that may be present into the solution. *I*_p_ is proportional to the concentration of Cr(VI) in the solution.

In all experiments, for Cr(VI) with trace, the first measurement result must be discarded because it is unstable. The stripping voltammogram was recorded 3 times (*n* = 3), and the peak current (*I*_p_) and peak potential (*E*_p_) values are averaged from three repetitions.

The glassy carbon electrodes were cleaned by polishing the surface with fine Al_2_O_3_ powder (particle size 0.6 *μ*m) and then washed with distilled water and then with 1 M NaOH to remove all Al_2_O_3_ particles on the glassy carbon surface, and then the electrodes were dipped into 1 M HCl solution and finally washed with distilled water and the electrodes were dried with soft filter paper.

## 3. Results and Discussion

### 3.1. Differential Pulse Adsorptive Stripping Voltammetry (DP-AdSV) Using *In Situ* BiFE

In order to select the appropriate conditions for the method, the experimental conditions were fixed as shown in Table S1. A univariate method is applied to examine the effect of factors. The magnitude of the stripping peak current (*I*_p_) and the relative standard deviation of the *I*_p_ (RSD) are used for selecting the appropriate test conditions.

#### 3.1.1. Effect of Acetate Buffer Concentration

**Figure 1:**
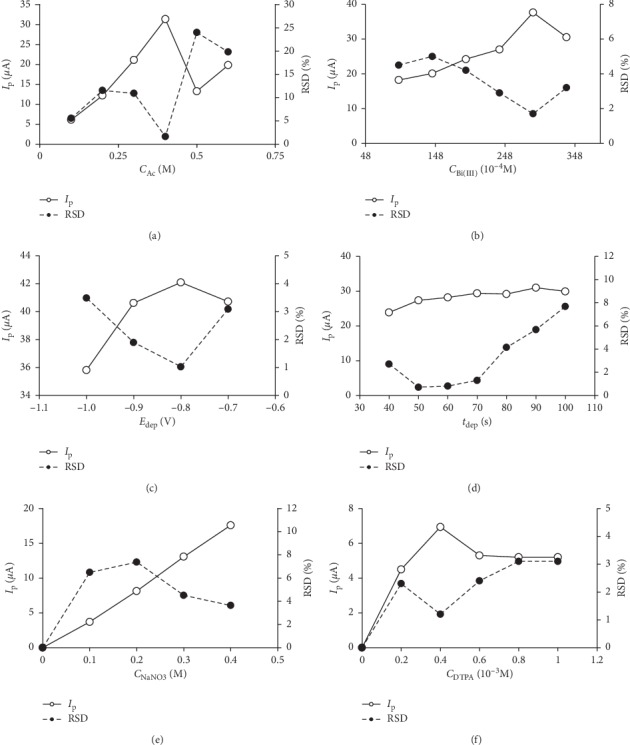
Effect of acetate buffer concentration (a), Bi(III) concentration (b), deposition potential (c), deposition time (d), NaNO_3_ concentration (e), and DTPA concentration (f) on the *I*_p_ of chromium in DP-AdSV using *in situ* BiFE. Experimental conditions are as mentioned in Sections 3.1.1to 3.1.4, and other conditions are as shown in Table S1.

Acetate buffer is chosen to stabilize the pH of the solution. Acetate buffer is one of the factors that strongly influence the complex of Cr(III) and DTPA [10]. The complex formation between Cr(III) and DTPA usually occurs at pH = 6 [10, 11]. At chromium concentration *C*_Cr(VI)_ = 3.8.10^−8^ M, ligand concentration *C*_DTPA_ = 0.4.10^−3^ M, and bismuth concentration *C*_Bi(III)_ = 24.10^−5^ M, the survey results of the effect of acetate buffer concentration (*C*_Ac_) in the range of 0.1 M–0.6 M (pH = 6) showed that *C*_Ac_ = 0.4 M was appropriate. With this condition, the peak current is 31.36 *μ*A and the repeatability is relatively good (RSD = 1.6% with *n* = 3) (Figure 1(a)).

#### 3.1.2. Effect of Bi(III) Concentration and DTPA Concentration

Previous studies with *ex situ* BiFE electrodes have suggested that the presence of KBr in the solution increases the bismuth's sustainability on the glassy disk surface and at the same time improves the conductivity of the solution [48]. In the acetate buffer with *C*_Ac_ = 0.4 M and the presence of KBr at a concentration of 5.0.10^−6^ M, *C*_DTPA_ = 0.4.10^−3^ M, and *C*_Cr(VI)_ = 3.8.10^−8^ M, a Bi(III) concentration of 28.8.10^−5^ M is appropriate. At those concentrations, the peak current is 37.6 *μ*A and the repeatability is good (RSD = 1.7% with *n* = 3) (Figure 1(b)). The survey results of the effect of DTPA concentrations in the range of 0.1 to 1.0.10^−3^ M to Cr peak current show that DTPA concentration of 0.4.10^−3^ M was appropriate (Figure 1(f)).

#### 3.1.3. Effect of NaNO_3_ Concentration

**Figure 2:**
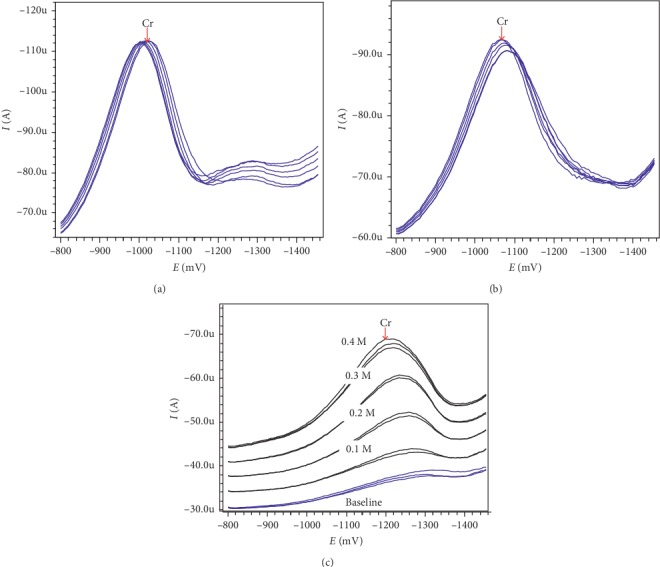
The stripping voltammetry DP-AdSV using *in situ* BiFE of chromium is recorded for (a) *E*_dep_ = −800 mV and (b) *t*_dep_ = 50 s. (c) NaNO_3_ concentrations are changed: the bottom line is the baseline, followed by concentration of NaNO_3_ increasing from 0.1 to 0.4 M. Other experimental conditions are as shown in Figure 1.

In the presence of NO_3_^−^, peak current of chromium (*I*_p_) was enhanced significantly. Many authors argue that NO_3_^−^ ions act as oxidizing agents that convert Cr(II)-DTPA complexes to Cr(III)-DTPA complexes and thus increase the concentration of Cr(III) on the electrode surface, and this leads to an increase in *I*_p_ [10, 11, 21]. At *C*_Ac_ = 0.4 M, *C*_KBr_ = 5.0.10^−6^ M, *C*_DTPA_ = 0.4.10^−3^ M, *C*_Bi(III)_ = 28.8 .10^−5^ M, and *C*_Cr(VI)_ = 3.8.10^−8^ M, *I*_p_ increased when the NaNO_3_ concentration increased from 0.1 M to 0.4 M (Figures 1(e) and 2(c)). However, when the NaNO_3_ concentration is greater than 0.4 M, it increases the baseline and may contaminate the analysis solution. When the NaNO_3_ concentration is equal to 0.4 M, the peak current is 17.6 *µ*A and the repeatability is quite good (RSD = 3.7% with *n* = 2). NaNO_3_ concentration value of 0.4 M was selected for further investigation.

#### 3.1.4. Effect of Deposition Potential (*E*_dep_) and Deposition Time (*t*_dep_)

When *C*_Ac_ = 0.4 M, *C*_KBr_ = 5.0.10^−6^ M, *C*_Cr(VI)_ = 2 ppb, *C*_DTPA_ = 0.4.10^−3^ M, and *C*_Bi(III)_ = 28.8.10^−5^ M, the survey results of the effect of the deposition potential in the range from −700 mV to −1000 mV are shown in Figure 1(c) and the stripping voltammetry is shown in Figure 2(a).

From this result, it shows that when *E*_dep_ is equal to −800 mV, the peak current is 42.1 *µ*A and the repeatability is good (RSD = 1.3% with *n* = 3). *E*_*d*ep_ of −800 mV was selected for further studies.

With the above conditions and when *E*_dep_ is equal to −800 mV, *I*_p_ is almost unchanged when the deposition time (*t*_dep_) is greater than 80 s, which means that it tends to reach saturation (Figure 1(d)). *t*_dep_ of 50 s is selected for the next experiment (the stripping voltammetry at *t*_dep_ = 50 s is shown in Figure 2(b)).

#### 3.1.5. Effect of Rotating Rate of Electrode (*ω*) and Equilibration Time (*t*_equal_)

By increasing the rotation speed of the electrode to a specified value, it will increase the mass transfer and the efficiency of the enrichment will be better. The survey results of the rotating rate of the electrode in the range from 800 rpm to 2400 rpm showed that *ω* of 2000 rpm was appropriate. At the end of the enrichment phase, the electrode should not be rotated for a specified period of time to keep the solution quiet and the electrode surface is stabilized (this time is also called equilibration time, symbolized as *t*_equal_). *I*_p_ survey results according to *t*_equal_ showed that *t*_equal_ of 50 s was appropriate.

#### 3.1.6. Effect of Cleaning Potential (*E*_clean_) and Cleaning Time (*t*_clean_)

The cleaning of the surface of the carbon glassy disc electrode at the end of each stripping voltammetry is essential, as it will create repeating electrode surfaces for subsequent measurements. In terms of experimental conditions as in Section 3.1.3, the survey results of the influence of *E*_clean_ in the range of 200 mV to 500 mV and *t*_clean_ in the range of 60 s to 120 s showed that *E*_clean_ is equal to 300 mV and *t*_clean_ is equal to 110 s which are appropriate.

### 3.2. Square Wave Adsorptive Stripping Voltammetry (SqW-AdSV) Using *In Situ* BiFE

Some authors argue that, in addition to differential pulse stripping voltammetry techniques, square wave stripping voltammetry can be used to record the signal (at this time, the method is called square wave adsorptive stripping voltammetry (SqW-AdSV)) and also allow the determination of very sensitive chromium. Based on the experimental conditions initially fixed as shown in Table S1, the effects of the factors were investigated in a similar way to the DP-AdSV method, and we obtained the appropriate conditions for SqW-AdSV (using *in situ* BiFE) to determine Cr(VI) as shown in Table S2.

### 3.3. Interferences

Interferences for the determination of Cr(VI) consist of metallic cations that have the stripping peak current near the stripping peak current of chromium and anions that can form complexes or make conjugates with the forms of chromium and Bi(III) which can be adsorbed onto the surface of the *in situ* BiFE, and surfactants can be adsorbed onto the working surface of the electrode.

The influence of interferences can be estimated by relative error values of stripping peak current (RE). Consider that RE for *I*_p_ was equal to RE for *C* (because *I*_p_ = kC). RE for *I*_p_ (or C) was accepted when it was equal to ½ Horwitz function RSD (RE *I*_p(Cr)_ ≤ ½ RSD_Horwitz_ = ½.2^(1−0.5lgC)^ = 32% with *C* = 0.2 ppb). RE was calculated as follows:

(3) $$\begin{array}{c} \mathrm{RE}\;I_{\text{p}}\left(\text{Cr}\right)\left(\%\right)=\frac{\left[I_{\text{p}}\left(\text{Cr}\right)-I_{\text{p}}{\left(\text{Cr}\right)}^{0}\right]}{I_{\text{p}}\left(\text{Cr}\right)0}*100, \end{array}$$

where RE is the relative error values of stripping peak current, *I*_p_ (Cr)^0^ is the stripping peak current without adding interferences and *I*_p_(Cr) is the stripping peak current with adding interferences.

#### 3.3.1. Interference Studies

When the chromium(III) concentration is about 100 times higher than the chromium(VI) concentration, the *I*_p_ does not change significantly (RE < 18%). In fact, rarely encountered C_Cr(III)_ case is 300 times higher than C_Cr(VI)_, so it can be assumed that Cr(III) does not affect the Cr(VI) determination. This investigation again confirms that Cr(III) does not affect the determination of Cr(VI) (Table S3 and Figure S3).

In the acetate buffer (pH = 5–6), Zn(II), Co(II), and Ni(II) can affect the determination of Cr(VI) because it has a stripping peak current close to the stripping peak current of Cr(VI). *E*_p (Zn)_ ≈ −1040 ÷ −1050 mV, *E*_p_ (Co) ≈ −1290 ÷ −1300 mV, *E*_p_ (Ni) ≈ −1080 ÷ −1100 mV, and *E*_p_ (Cr) ≈ −1180 ÷ −1240 mV.

From the experimental results at approximately 3.8 10^−9^ M (0.2 ppb) chromium concentration, 120 s deposition time, and the suitable conditions as in Table 2, we have seen that Zn has not influenced the determination of chromium when Zn(II) concentration is 800 times larger than Cr(VI) concentration (RE_Ip(Cr)_ ≤ 16%) (Table 2), and in consequence, we can determine chromium in the natural water with attendance of Zn(II) because ordinarily Zn(II) concentration is 500 times smaller than Cr(VI) concentration in natural water (Table 2). Co and Ni have not influenced the determination of chromium when Co(II) and Ni(II) concentrations are 90 times larger than Cr(VI) concentration (RE *I*_p(Cr)_ = 1.7–8.0% for Co(II) and 3.9–19.4% for Ni(II) (Table 2).

In natural water, Fe(III) and Ca(II) usually exist at high concentrations of mM; in seawater, Ca(II) exists at quite high concentration, about 10^−2^ ÷ 10^−3^ M, so it is necessary to examine the effect of Fe(III) and Ca(II) on the stripping peak current of Cr(VI). The results in Table S4 show that when the concentration of Fe(III) and Ca(II) increases to 36.10^−6^ M and 50.10^−6^ M, respectively, meaning that the concentrations of Fe(III) and Ca(II) are about 10,000 times greater than the concentration of Cr(VI), the determination of Cr(VI) is not affected. RE is less than 17%.

In natural water, Cl^−^ and SO_4_^2−^ ions have significant concentrations (mM and larger), and they can form complexes with metals present in the study solution and may therefore affect the determination of Cr(VI). To investigate the effects of Cl^−^ and SO_4_^2−^, a series of experiments were conducted with Cl^−^ concentrations ranging from 0 to 281.7.10^−3^ M and SO_4_^2−^ concentrations ranging from 0 to 10.4.10^−3^ M. The results in Table S5 show that Cl^−^ does not affect the determination of Cr(VI) when *C*_Cl−_ is in the range of 0 to 14.09. 10^−3^ M. When *C*_Cl−_ is greater than 28.17.10^−3^ M (nearly equivalent to Cl^−^ concentration in brackish water), Cl^−^ affects the determination of Cr(VI) with RE > 32%. Therefore, when analyzing Cr(VI) in samples with high Cl^−^concentration, it is necessary to take Cl^−^ removal method from the sample. SO_4_^2−^ did not affect the determination of Cr(VI) by SqW-AdSV/*in situ* BiFE method with RE < 27%.

In the adsorption stripping voltammetry method, the surfactant can be adsorptive on the surface of the working electrode, and this can affect the adsorption process of the metallic complexes on the working electrode. Triton X-100 (polyethylene glycol) is a typical nonionic surfactant and usually is used in order to observe the influence of the surfactant on the adsorption stripping voltammetry method. The effects of Triton X-100 are investigated at concentrations between 0 and 93. 10^−9^ M, and the results in Table S6 show that, when increasing the concentration of Triton X-100 to 25 times higher than the concentration of Cr(VI), it still did not affect the determination of Cr(VI) with RE < 8%.

In fact, the concentration of natural surfactants is rarely greater than 77.10^−9^ M, and therefore, it can be assumed that they do not affect the Cr(VI) determination. Thus, when determining Cr(VI) by the adsorption stripping voltammetry method, it is not necessary to remove the surfactant from the analytical solution.

In some cases, natural water and wastewater contain many organic substances including surfactants. It is necessary to treat the sample to exclude organic substances before analysis using UV irradiation and decomposition in acid mixture.

### 3.4. Evaluation of Reliability of DP-AdSV and SqW-AdSV Methods

#### 3.4.1. Repeatability

**Figure 3:**
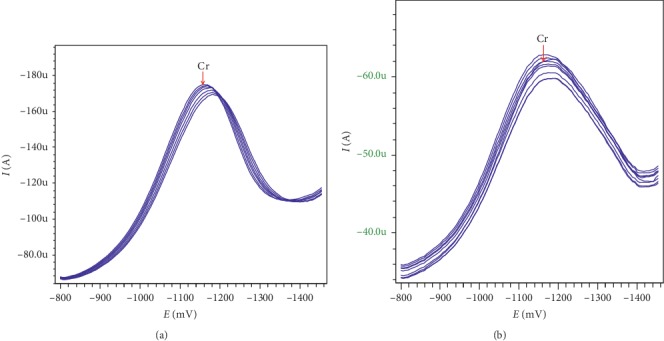
The stripping voltammetry lines were repeated: (a) according to the SqW-AdSV method (*n* = 7) with *C*_Cr(VI)_ = 1 ppb; (b) according to the DP-AdSV method (*n* = 9) with *C*_Cr(VI)_ = 2 ppb. Other experimental conditions are as shown in Table S2.

Repeat recording of 7 stripping voltammetry lines (*n* = 7) on the same *in situ* BiFE according to the DP-AdSV or SqW-AdSV method in Figure 3 shows that *I*_p_ in both approaches has good repeatability with RSD < 4% (*n* = 9) and RSD < 3% (*n* = 7), respectively, for DP-AdSV and SqW-AdSV. The stripping peak of chromium (*E*_p_) is negligible, only about 20 mV toward the positive side.

#### 3.4.2. Linear Range and Detection Limits

**Figure 4:**
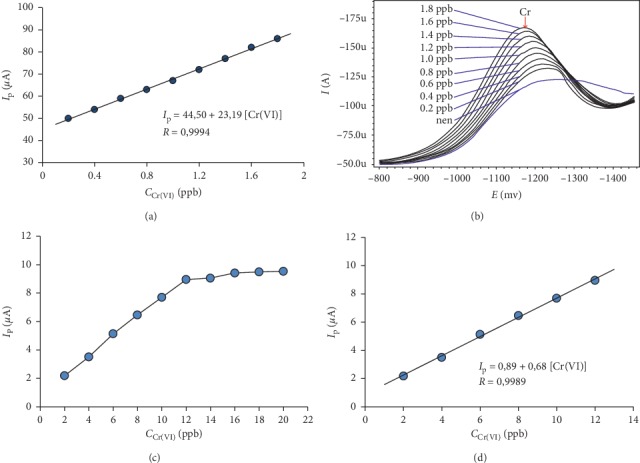
(a) Linear regression line for the SqW-AdSV method; (b) stripping voltammetry of SqW-AdSV method: the bottom line is the baseline, followed by nine additional standard lines, each adding 0.2 ppb; (c) relation between *I*_p_ and *C*_Cr(VI)_ when examining the linear range of the DP-AdSV method; (d) linear regression line for the DP-AdSV method. Experimental conditions are as shown in Table S2.

The linear range and LOD of the two methods SqW-AdSV and DP-AdSV were investigated with the appropriate experimental conditions as shown in Table S2 and the stripping voltammetry specifications as shown in Table S1, and the following results were obtained:

*(1) The Linear Range*. For the SqW-AdSV method, *I*_p_ and *C*_Cr(VI)_ have a good linear correlation in the range *C*_Cr(VI)_ = 0.3 ÷ 1.8 ppb with a correlation coefficient (*R*) of 0. 9994 (linear regression equation is shown in Figure 4(a), and the stripping voltammetry is shown in Figure 4(b)).

For the DP-AdSV method, there is a good linear correlation in the range *C*_Cr(VI)_ = 2 ÷ 12 ppb with *R* = 0.9989 (linear regression equation is shown in Figure 4(d)).

*(2) Sensitivity*. The SqW-AdSV method achieved a sensitivity (23 *µ*A/ppb) of about 34 times higher than the DP-AdSV (0.682 *µ*A/ppb) method.

*(3) Detection Limits and Quantitative Limits*. For SqW-AdSV (when *E*_dep_ = −800 mV and *t*_dep_ = 160 s): LOD = 0.1 ppb; LOQ = 0.3 ppb. For DP-AdSV (when *E*_dep_ = −800 mV and *t*_dep_ = 50 s): LOD = 0.6 ppb; LOQ = 2 ppb.

Thus, the SqW-AdSV method achieves a narrower linear range than the DP-AdSV method, but it achieves higher sensitivity than the DP-AdSV method (due to its lower LOD and greater slope linearity). It can be said that with LOD as above, DP-AdSV and SqW-AdSV methods can be used with *in situ* BiFE to determine the trace amount of Cr(VI).

### 3.5. Determination of Chromium in Natural Water by the SqW-AdSV Using *In Situ* BiFE

In natural water samples, chromium usually exists in both Cr(VI) and Cr(III) forms. As investigated, the SqW-AdSV/*in situ* BiFE method identifies Cr(VI) and also determines the total Cr(VI) + Cr(III) if during the decomposition of the sample, an additional oxidizer is added to oxidize Cr(III) to Cr(VI). Thus, we can determine chromium in individual forms by determining Cr(VI) (^*∗*^) and total Cr(VI) + (III) (^*∗∗*^). It follows that the Cr(III) content is the difference of (^*∗∗*^) and (^*∗*^).

Based on the above results, it is possible to apply the SqW-AdSV/*in situ* BiFE to determine the trace of Cr(VI) with LOD ≈ 0.1 ppb. With that LOD, the SqW-AdSV/*in situ* BiFE can directly determine the amount of Cr(VI) in natural water, without the stage of getting rich, and this is a great advantage of the SqW-AdSV/BiFE method.

In order to answer the question of whether the analytical method used to analyze the amount of chromium in natural water samples can be applied, we have conducted experiments to verify the correctness (through the Certified Reference Material (CRM)) and an analysis of some natural water samples. On the basis of the experiments mentioned above, the analysis process of Cr(VI) and total Cr(III, VI) in natural water by the SqW-AdSV method was proposed.

#### 3.5.1. Quality Control of Analytical Methods through Standard Sample Analysis

In order to confirm the practical applicability of the SqW-AdSV method to analyze chromium traces using BiFE electrodes, it is necessary to control the analytical method quality by evaluating the accuracy and repeatability when analyzing standard samples.

*(1) For Surface Water Samples*. Surface water Certified Material Reference (SPS-SW1 Batch 122) was selected to evaluate the accuracy of the method. The actual value of the chromium content of the sample is 2.00 ± 0.02 ppb (95% confidence boundary *e* = ± 0.02 ppb). Analysis of standard SPS-SW1 surface water (CRM) samples by the SqW-AdSV using *in situ* BiFE with the appropriate experimental conditions is shown in Table 3. The analysis was repeated 3 times. The volume of the solution to be charged to the electrolyser is 2 mL, and the volume of solution in the electrolyser is 10 mL.

The results in Table 4 show that the SqW-AdSV/*in situ* BiFE has good repeatability (the standard deviation is 4% for the repetition of 3, and it is less than half the standard deviation based on the Horwitz function (RSD_H_ = 2^(1−0,5lgC)^; when the chromium concentration is 2 ppb, the standard deviation of the Horwitz equation is 41%) [49] and the method has good accuracy because the chromium content is within the 95% confidence interval of the CRM sample.

*(2) For Seawater Samples*. Analysis of the standard seawater CRM coded NASS 6 by the SqW-AdSV/*in situ* BiFE with the appropriate experimental conditions as shown in Table 3.

Because the concentration of chromium in NASS 6 seawater was too small to be directly analyzed, only NASS 6 standard sample was used as the matrix for analysis and validity. The actual value of the chromium content in the NASS 6 sample is 0.116 ± 0.008 ppb (the 95% confidence bound *e* = ± 0.008 ppb). NAAS 6 standard sample was added with standard Cr(VI) solution to attain 3 levels of 2 ppb, 6 ppb, and 10 ppb and then analyzed with the standard added samples to determine recovery.

The results showed that the SqW-AdSV/*in situ* BiFE for chromium analysis in seawater samples has a good accuracy (recoverability from 94 to 109%). According to the AOAC (American Association of Analytical Chemists) when analyzing the levels of 1.0 to 10 ppb, achieving a recovery rate of 80 to 110% is acceptable [50]. Therefore, it is possible to use this method to analyze chromium in seawater samples (Table 5).

The results of the linear range, sensitivity, limit of detection, and accuracy showed that it is possible to use SqW-AdSV/*in situ* BiFE to determine chromium in surface water and seawater.

#### 3.5.2. Real Sample Analysis

For the purpose of testing the possibility of applying the SqW-AdSV/*in situ* BiFE method for the analysis of chromium in water environment, well water, tap water, lagoon water, and seawater in some different areas in Thua Thien Hue province were taken for analysis.

Water samples were taken in clean PET bottles and acidified with concentrated HCl (500 *μ*l HCl/500 ml of sample). Samples were filtered through 0.45 *μ*m porous fiberglass filter paper and analyzed immediately after filtration.

Collected and stored samples were analyzed directly (after filtration through a 0.45 *μ*m porous fiberglass filter paper) to determine the Cr(VI) content by the SqW-AdSV/*in situ* BiFE; the total chromium content of the sample was determined after the decomposition of the sample by the method of (a) ((a): add 50 *μ*l of concentrated HCl, 25 *μ*l of H_2_O_2_ 35% to 50 ml of sample in the Teflon cup, boil for 90 minutes, let it cool, and adjust up to 25 ml) [51].

Samples were analyzed by SqW-AdSV/*in situ* BiFE according to the process shown in Figure S2. The results of actual sample analysis are presented in Table 6.

## 4. Conclusion

Using *in situ* BiFE electrodes with DTPA complexing ligands in acetate buffer solution pH 6 with the presence of KBr and NO_3_^−^ ion, DP-AdSV and SqW-AdSV methods can determine chromium(VI) concentrations of 0.3 ppb and 2.0 ppb, respectively. The proposed method has been successfully applied for chromium analysis in some natural water samples such as lagoon water, well water, tap water, and saltwater in some areas of Thua Thien Hue province, Vietnam. This *C*_Cr(VI+III)._ analysis procedure in water sample by SqW-AdSV/*in situ* BiFE method was satisfactorily applied for the determination of chromium in real water such as tap water, river water, and well water samples in all countries of the world. The determination of chromium in the above real water sample could be carried out within 60 min.

## Figures and Tables

**Table 1:** Summary of published works on stripping voltammetry method for chromium determination.

No.	Ligands	Background solution	Working electrode	Measurement techniques	LOD (pbb)	Analytical object	Determined form	Time of publication	References
1	DTPA	CH_3_COONH_4_, NaNO_3_ (pH = 5.2)	HMDE	DP-AdSV	1.2	Fake template	Total chromium	1987	[21]
2	Diphenylcarbazide	H_2_SO_4_ 0.1 M	GE	LSV	0.05	Fake template	Cr(VI)	1988	[29]
3	TTHA	CH_3_COONa, NaNO_3_ (pH = 5.5)	HMDE	DP-CSV	1.04	Fake template	Cr(VI)	1988	[29]
4	TTHA	CH_3_COONa, NaNO_3_ (pH = 5.5)	HMDE	SqW-AV	0.02	Fake template	Cr(VI)	1988	[29]
5	DTPA	KH_2_PO_4_, Na_2_HPO_4_ (pH = 6–7)	HMDE	DP-CSV	0.01	Fake template	Cr(VI)	1990	[32]
6	Diphenylcarbazide	H_2_SO_4_ 0.3 M	HMDE	DP-AdSV	0.02	Groundwater	Cr(VI)	1992	[23]
7	DTPA 2.5 mM	Acetate buffer (pH 5.2)	HMDE	SqW-AdSV	0.05	Seawater	Cr(VI), total chromium	1992	[45]
8	DTPA 0.01 M	NaNO_3_ 0.5 M, MES (morpholinoethanesulfonic acid) (pH 6.1)	HMDE	DP-AdSV	0.62	River water, tap water	Total chromium	2000	[28]
9	DTPA	NaNO_3_, acetate buffer (pH 5.7)	HMDE	DP-AdSV	Cr(VI): 0.005 activated Cr(III): 0.52	River water, lake water, sewer water	Cr(VI), activated Cr(III), total chromium	2001	[1]
10	TTHA 0.1 M (triethylenetetraminhexaacetic acid)	NaCH_3_COO 1 M, NaNO_3_ 5 M (pH 6.2–6.5)	SMDE	SqW-AdSV	0.52	Leaf	Cr(VI)	2003	[19]
11	DTPA	Acetate buffer (pH = 6), KNO_3_ 0.25 M	HMDE	DP-AdSV	0.004	Wastewater	Cr(VI), total chromium	2004	[22]
12	Rubeanic acid (dithiooxamide)	Acetate buffer (pH 6), KNO_3_ 0.20 M	HMDE	DP-AdSV	1976	River water, seawater, sewage, vinegar	Cr(VI)	2012	[33]
13	DTPA	Acetate buffer (pH 6) NaNO_3_	HMDE	AdSV	18.2	Human urine	Cr(VI)	2005	[26]
14	TTHA 0.2 M	NaCH_3_COO 0.1 M, NaNO_3_ 5 M (pH 6.2)	HMDE	CV-AdSV	0.3		Cr(VI)	1997	[31]
15	DTPA	NaCH_3_COO 0.01 M, NaNO_3_ 0.5 M (pH 8.5)	SMDE		156.10^−5^	River water, tap water	Cr(VI)	1999	[35]
16	DTPA 5 mM	NaCH_3_COO 0.15 M, NaNO_3_ 0.7 M (pH 6)	HMDE	SqW-AdSV	0.05	Cement	Cr(VI)	2011	[25]
17	Pyrogallol red	0.4 M acetate buffer (pH 4.5)	HMDE	SqW-AdSV	0.05	Seawater	Cr(VI), Cr(III), total chromium	2012	[34]
18	DTPA 0.05 M	NaCH_3_COO 0.2 M (pH 6.2)	HMDE	DP-AdSV	1.04	Electroplating waste water	Cr(VI)	2004	[27]
19	Pyrocatechol violet	Acetate buffer	HMDE	DP-AdSV			Cr(VI)	1997	[24]
20	HEDTA (N-2-hydroxyethyl ethylenediamine-N,N′,N″-triacetic acid) and PCV (pyrocatechol violet)	0.1 M acetate buffer (pH 6) KNO_3_ 2M	HMDE	DP-AdSV			Cr(III), Cr(VI)	2002	[30]
21	Cupferron 0.01 M	PIPES 0.2 M (pH = 7)	BiFE ex situ	SqW-AdSV	0.1	Laboratory water, cigarettes, soil sample	Total chromium	2004	[14]
22	DTPA 5 mM	Acetate 0.1 M (pH = 6.0)	BiFE ex situ	SqW-AdSV	0.015	River water	Total chromium	2005	[11]
23	DTPA 5 mM	NaOAc 0.1 M (pH = 6.0)	BiFE ex situ	SqW-AdSV	0.015	Blood sample	Total chromium	2006	[10]
24	DTPA 5 mM	Acetate 0.1 M (pH = 6.0)	BiFE ex situ	SqW-AdSV	0.017 (Cr^VI^); 0.022 (Cr total)	River water	Cr(VI); Cr(III)	2010	[16]
25	PAR (4-(2-pyridylazo) resorcinol)	CH_3_COOH–CH_3_COONa, trisodium citrate	BiFE ex situ	SqW-AdSV	0.01	Tap water, lake water, soil sample	Total chromium	2013	[17]
26	DTPA	CH_3_COONH_4_, NaNO_3_ (pH = 5.2)	Bi film wrapped single walled carbon nanotubes	DP-AdSV	0.12. 10^−3^	Fake template	Total chromium	2013	[18]
27	DTPA 0.01 M	0.1 M acetate buffer (pH = 6.0)	Hg (Ag)FE	DP-AdSV	0.004	Natural water, drinking water	Cr(VI)	2006	[12]
28	TTHA	CH_3_COONa, NaNO_3_ (pH = 5.5)	Gold film modified carbon composite electrode	DP-CSV	4.0	Fake template	Cr(VI)	2014	[37]
29	PET (4pyridine-ethanethiol)	NaF 0.15 M (pH = 4.5)	PET/nano-Au/Pt-RD electrode	DP-AdSV	0.001	Seawater	Cr(VI)	2015	[13]
30	DTPA	KH_2_PO_4_, Na_2_HPO_4_ (pH = 6–7)	(Flower-like self-assembly of gold nanoparticles) AuNPs/GCE	DP–CSV	0.001	Fake template	Cr(VI)	2012	[38]
31		HCl (pH = 2)	AuNPs/nano-TiC/GCE	DP-AdSV	2.08	Seawater	Cr(VI)	2015	[15]
32		HClO_4_ 0.06 M	AuNPs/SPCE	ASV	0.002	Tap water, seawater	Cr(VI)	2015	[39]
33	QH2 (quercetin)	Acetate buffer (pH = 6), KNO_3_ 0.7 M	QH2/MWCNT-SPCE (quercetin/multiwalled carbon nanotubes screen-printed carbon electrode)	DP-AdSV	15.9	Drinking water	Cr(VI)	2013	[20]
34	-	Acetate buffer (pH = 5)	*μ* NPs/GCE	DP-SV	0.01	Electroplating waste water	Cr(III)	2015	[44]
35	DTPA 0.1 M	Acetate buffer (pH 6), KNO_3_ 0.25 M	*μ* NPs/BiFE	DP-AdSV	0.12. 10^−3^	Fake template	Cr(VI)	2011	[36]
36	Polyvinyl butyral/SPEs + 4.7% DTPA	H_2_SO_4_ (pH 1)	SPEs (screen-printed electrode)	CV-AdSV	52.0	Fake template	Cr(VI)	2014	[42]
37	2,5,8,11,14-Pentaaza-15,16,29- phenanthrolinophane (NeoTT)	1,6-Dichloro-hexane (DCH), LiCl 10 mM, HCl 1 mM	Liquid/liquid interface	SqW-AdSV	250.0	Fake template	Cr(VI)	2005	[40]
38		Silver: AgClO_4_ 0.1 mM, briton robinson (pH 2) gold: HAuClO_4_ 0.1 mM, H_2_SO_4_ 0.5 mM.	Carbon screen- printed electrode (CSPEs)	DPV	Silver: 44.2 gold: 20.8	Fake template	Cr(VI)	2008	[43]
39	Septonex 10^−6^M (1-pentadecyltrimethylamonium bromide)	HCl 0.25 M, NaCl 0.1 M (pH < 2)	Carbon paste	DP-CSV	2.6	Tea	CrO_4_^2−^	2004	[41]

**Table 2:** Influence of Zn(II), Co(II), and Ni(II) concentrations on peak current.

Cation	Zn(II)			Co (II)			Ni (II)		
No.	*C*_Zn(II)_(nM)	*I*_p_(Cr)(*μ*A)	RE *I*_p(Cr)_(%)	*C*_Co(II)_(nM)	*I*_p_(Cr)(*μ*A)	RE_Ip(Cr)_(%)	*C*_Ni(II)_(nM)	*I*_p_(Cr)(*μ*A)	RE *I*_p(Cr)_(%)
1	0	96.4	0	0	76.5	0	0	49.7	0
2	770	96.3	0.1	84	82.4	7.6	84	53.2	3.9
3	1540	94.6	1.9	168	82.5	7.9	168	58.3	10.4
4	2310	87.9	8.7	252	81.7	6.8	252	61.5	14.6
5	3080	80.9	16.0	336	77.8	1.7	336	62.4	19.4

Conditions: *C*_Cr(VI)_ = 3.8.10^−9^ M = 0.2 ppb; *C*_Bi(III)_ = 28.8.10^−5^ M; *t*_ad_ = 120 s; *E*_clean_ = 400 mV; *t*_clean_ = 100 s; *U*_step_ = 6 mV; *v* = 210 mV/s; ∆*E* = 30 mV; *f* = 35 Hz.; *ω*  = 2000 rpm; *C*_DTPA_ = 0.4 mM; *C*_Ac_ = 0.4 M; *C*_NaNO3_ = 0.4 M; *E*_ad_ = −800 mV.

**Table 3:** Appropriate experimental conditions for the SqW-AdSV/*in situ* BiFE for the determination of Cr(VI).

No.	Parameter (unit of measure)	Symbol	SqW-AdSV/*in situ* BiE
1	DTPA concentration (M)	*C*_DTPA_	0.4. 10^−3^
2	Concentration of acetate buffer (pH = 6) (M)	*C*_Ac_	0.40
3	NaNO_3_ concentration (M)	*C*_NaNO3_	0.40
4	KBr concentration (M)	*C*_KBr_	5.10^−6^
5	Bi(III) concentration (M)	*C*_Bi(III)_	28.8. 10^−5^
6	Cleaning potential (mV)	*E*_clean_	300
7	Cleaning time (s)	*t*_clean_	100
8	Rotating speed of working electrode (rpm)	(*ω*) 2000	2000
9	Deposition potential (mV)	*E*_dep_	-800
10	Deposition time (s)	t_dep_	200
11	Equilibration time (s)	*t*_equal_	50
12	Potential sweep range (mV)	*E*_range_	−800 ÷ −1450
13	Technical parameters		
	•Amplitude (mV)	∆*E*	30
	•Voltage step (mV)	*U*_step_	6
	• Sweep rate (mV/s)	*v*	210
	• Frequency (Hz)	*f*	35

**Table 4:** Accuracy of the SqW-AdSV/*in situ* BiFE for the determination of chromium in surface water.

Information		[Cr(VI)](ppb)	C_Cr_ (ppb)
Experiment	1	0.38	1.90
	2	0.40	2.00
	3	0.40	2.00
Average ± S (ppb)		1.97 ± 0.08	
Cr content in the CRM sample (ppb)		2.00 ± 0.02 (*C*_Cr_ = 1.98 ÷ 2.02 ppb)	
RSD (%), *n* = 3		4	

^(a)^[Cr(VI)] is the concentration of Cr(VI) in the electrolyte minus the blank. White sample has [Cr(VI)] = 0.034 ppb; C_Cr_ is the Cr content in the sample (calculated by the formula: *C*_Cr_ = [Cr(VI)] .*V*_2_/*V*_1_). *V*_1_: volume of solution taken into the electrolyser (*V*_1_ = 2 ml), *V*_2_: volume of solution in the electrolyser (*V*_2_ = 10 mL), S is the standard deviation. Experimental conditions are as shown in Table 3.

**Table 5:** Determination of the accuracy of the SqW-AdSV/*in situ* BiFE on the NASS 6^a^.

[NAAS 6]	The content of chromium in the sample (ppb) ×1	Chromium standard added (ppb)×o	The content of chromium in standard added samples (ppb) ×2	Recovery (%)
2 ppb	0.116	1.884	1.915	96
			2.043	102
			1.941	97
Average ± S			1.966 ± 0.054	

6 ppb	0.116	5.884	5.958	99
			6.258	104
			5,655	94
Average ± S			5.957 ± 0.213	

10 ppb	0.116	9.884	10.687	107
			10.125	101
			10.887	109
Average ± S			10.566 ± 0.279	

^a^Recovery = (x_2_−x_1_).·100/*x*_0_; S is the standard deviation; experimental conditions are as shown in Table 3.

**Table 6:** Chromium content in lagoon water samples, tap water, well water, and saltwater.

No	Sample type	Sample symbol	Chromium concentration (*C*_mean_ ± *ε*) ppb, *n* = 3, *P* = 0.95	
			*C*_Cr(VI + III)_	*C*_Cr(VI)_
1	Water sample of Cau Hai Lagoon	M1	13.8 ± 0.2	1.0 ± 0.1
2		M2	19.0 ± 1.0	1.0 ± 0.2
3		M3	7.3 ± 0.4	1.5 ± 0.2
4		M4	26.1 ± 5.8	1.6 ± 0.3
5		M5	14.1 ± 0.8	1.3 ± 0.7
6		M6	1.0 ± 0.1	0.7 ± 0.2
7		M7	11.1 ± 4.4	0.8 ± 0.3

8	Tap water	PTN	20.0 ± 2.3	
9		GĐ	19.2 ± 3.3	
10		GĐ1	18.1 ± 0.8	

11	Well water	G1	28.6 ± 1.0	
12		G1′	24.6 ± 4.0	
13		G2	22.3 ± 4.2	
14		G2′	12.5 ± 4.8	
15		G3	6.4 ± 0.6	
16		G3′	14.4 ± 0.5	
17		G4	13.6 ± 3.3	
18		G4′	21.2 ± 4.4	

19	Saline water	B1	1.3 ± 0.3	1.0 ± 0.2
20		B2	16.1 ± 1.3	12.1 ± 1.4

## Data Availability

The data used to support the findings of this study are available from the corresponding author upon request.

## Supplementary Materials

Supplementary Materials Table S1: for the DP-AdSV and SqW-AdSV using in situ BiFE. Table S2: suitable experimental conditions for DP-AdSV and SqW-AdSV methods using in situ BiFE. Table S3: influence of chromium(III). Figure S1: SqW-AdSV/BiFE in situ stripping voltammograms of chromium(VI) when examining the effects of chromium(III). Table S4: influence of Fe(III) and Ca(II). Table S5: influence of Cl^−^ and SO_4_^2−^. Table S6: influence of Triton X-100. Figure S2: diagram of *C*_Cr_(VI + III) analysis procedure in water sample by SqW-AdSV/in situ BiFE method. .

## References

[1] Li Y., Xue H. (2001). Determination of Cr (III) and Cr (VI) species in natural waters by catalytic cathodic stripping voltammetry. *Analytica Chimica Acta*.

[2] Bobrowski A., Królicka A., Zarębski J. (2009). Characteristics of voltammetric determination and speciation of chromium—a review. *Electroanalysis*.

[3] James B. R., Petura J. C., Vitale R. J., Mussoline G. R. (1995). Hexavalent chromium extraction from soils: a comparison of five methods. *Environmental Science & Technology*.

[4] Vitale R. J., Mussoline G. R., Petura J. C., James B. R. (1994). Hexavalent chromium extraction from soils: evaluation of an alkaline digestion method. *Journal of Environmental Quality*.

[5] Divrikli U., Soylak M., Elci L. (2008). Determination of total chromium by flame atomic absorption spectrometry after coprecipitation by cerium (IV) hydroxide. *Environmental Monitoring and Assessment*.

[6] Yao L., Zhu Y., Xu W. (2019). Combination of dispersive solid phase extraction with dispersive liquid-liquid microextraction for the sequential speciation and preconcentration of Cr (III) and Cr (VI) in water samples prior to graphite furnace atomic absorption spectrometry determination. *Journal of Industrial and Engineering Chemistry*.

[7] Sereshti H., Khojeh V., Samadi S. (2011). Optimization of dispersive liquid-liquid microextraction coupled with inductively coupled plasma-optical emission spectrometry with the aid of experimental design for simultaneous determination of heavy metals in natural waters. *Talanta*.

[8] Zhu Q.-y., Zhao L.-y., Sheng D. (2019). Speciation analysis of chromium by carboxylic group functionalized mesoporous silica with inductively coupled plasma mass spectrometry. *Talanta*.

[9] Laghari A. J., Khuhawar M. Y., Zardari L. A. (2007). Capillary gas chromatographic analysis of pyrrolidinedithiocarbamate metal chelates. *Journal of Separation Science*.

[10] Yong L., Armstrong K. C., Dansby-Sparks R. N., Carrington N. A., Chambers J. Q., Xue Z.-L. (2006). Quantitative Analysis of trace Chromium in blood samples. Combination of the advanced oxidation Process with catalytic adsorptive stripping voltammetry. *Analytical Chemistry*.

[11] Lin L., Lawrence N. S., Thongngamdee S., Wang J., Wang Y., Lin Y. (2005). Catalytic adsorptive stripping determination of trace chromium (VI) at the bismusth film electrode. *Talanta*.

[12] Bas’ B. (2006). Refreshable mercury film silver based electrode for determination of chromium (VI) using catalytic adsorptive stripping voltammetry. *Analytica Chimica Acta*.

[13] Du N. H., Luyen N. T. T., Manh N. K. (2015). Determination of ppt level chromium (VI) using the gold nano-flakes electrodeposited on platinum rotating disk electrode and modified with 4-thiopyridinium. *American Journal of Analytical Chemistry*.

[14] Chatzitheodorou E., Economou A., Voulgaropoulos A. (2004). Trace determination of chromium by square-wave adsorptive stripping voltammetry on bismuth film electrodes. *Electroanalysis*.

[15] Han H., Pan D., Liu D., Hu X. (2015). Cathodic stripping voltammetric determination of chromium in coastal waters on cubic Nano-titanium carbide loaded gold nanoparticles modified electrode. *Frontiers in Marine Science*.

[16] Jorge E. O., Rocha M. M., Fonseca I. T. E., Neto M. M. M. (2010). Studies on the stripping voltammetric determination and speciation of chromium at a rotating–disc bismuth film electrode. *Talanta*.

[17] Zhang Q., Zhong S.-wei, Su J.-li, Li X.-jun, Zou H. (2013). Determination of trace chromium by square-wave adsorptive cathodic stripping voltammetry at an improved bismuth Film Electrode. *The Electrochemical Society*.

[18] Ouyang R., Zhang W., Zhou S. (2013). Improved Bi film wrapped single walled carbon nanotubes for ultrasensitive electrochemical detection of trace Cr (VI). *ElectroChimica Acta*.

[19] Misiego A. S., Carra R. M. G., Carracedo P. A., Torre M. E. M. (2003). Determination of Cr (in small quantities) by adsorptive stripping voltammetry: a comparative study of square wave versus differential pulse. *Analytical and Bioanalytical Chemistry*.

[20] Sadeghi S., Garmroodi A. (2013). A highly sensitive and selective electrochemical sensor for determination of Cr (VI) in the presence of Cr (III) using modified multi-walled carbon nanotubes/quercetin screen-printed electrode. *Materials Science and Engineering: C*.

[21] Torrance K., Gatford C. (1987). Determination of soluble chromium in simulated P.W. R coolant by differential pulse adsorptive stripping voltammetry. *Talanta*.

[22] Bobrowski A., Ba´s B., Dominik J., Niewiara E. (2004). Chromium speciation study in polluted waters using catalytic adsorptive stripping voltammetry and tangential flow filtration. *Talanta*.

[23] Elleouet C., Quentel F., Madec C. (1992). Determination of trace amounts of chromium (VI) in water by electrochemical methods. *Analytica Chimica Acta*.

[24] Vukomanovic D. V., vanLoon G. W., Nakatsu K., Zoutman D. E. (1997). Determination of chromium (VI) and (III) by adsorptive stripping voltammetry with pyrocatechol violet. *Microchemical Journal*.

[25] Panaščikaitė E., Latvėnaitė I., Armalis S. (2011). Determination of chromium in cement by catalytic adsorptive stripping voltammetry. *Chemija*.

[26] Husakova L., Bobrowski A., Sramkova J., Krolicka A., Vytras K. (2005). Catalytic adsorptive stripping voltammetry versus electrothermal atomic absorption spectrometry in the determination of trace cobalt and chromium in human urine. *Talanta*.

[27] Kiptoo J., Ngila J. C., Sawula G. M. (2004). Speciation studies of nickel and chromium in wastewater from an electroplating plant. *Talanta*.

[28] Korolczuk M. (2000). How faster and cheaper to determine chromium by adsorptive cathodic stripping voltammetry in the presence of DTPA and nitrate. *Fresenius Journal of Analytical Chemistry*.

[29] Goldoni M., Caglieri A., Poli D. (2006). Determination of hexavalent chromium in exhaled breath condensate and environmental air among chrome plating workers. *Analytica Chimica Acta*.

[30] Dom´ınguez O., Julia Arcos M. (2002). Simultaneous determination of chromium (VI) and chromium (III) at trace levels by adsorptive stripping voltammetry. *Analytica Chimica Acta*.

[31] Palrecha M., Mathur P. K. (1997). Adsorptive stripping voltammetric determination of chromium in gallium. *Talanta*.

[32] Scholz F., Lange B., Draheim M., Pelzer J. (1990). The catalytic adsorptive stripping voltammetric determination of chromium with DTPA and nitrate. *Fresenius’ Journal of Analytical Chemistry*.

[33] Abbasi S., Bahiraei A. (2012). Ultra trace quantification of chromium (VI) in food and water samples by highly sensitive catalytic adsorptive stripping voltammetry with rubeanic acid. *Food Chemistry*.

[34] Arancibia V., Nagles E., Gómez M., Rojas C. (2012). Speciation of Cr (VI) and Cr (III) in water samples by adsorptive stripping voltammetry in the presence of pyrogallol red applying a selective accumulation potential. *International Journal of Electrochemical Science*.

[35] Korolczuk M., Grabarczyk M. (1999). Voltammetric determination of Cr (VI) in a flow system in the presence of diethylenetriaminepentaacetic acid (DTPA) following its deposition in the metallic state. *Analytica Chimica Acta*.

[36] Saturno J., Valera D., Carrero H., Fernández L. (2011). Electroanalytical detection of Pb, Cd and traces of Cr at micro/nano-structured bismuth film electrodes. *Sensors and Actuators B: Chemical*.

[37] Torabi Kachoosangi R., Compton R. G. (2014). Voltammetric determination of Chromium (VI) using a gold film modified carbon composite electrode. *Sensors and Actuators B*.

[38] Ouyang R., Bragg S. A., Chambers J. Q., Xue Z.-L., Xue Zi-L. (2012). Flower-like self-assembly of gold nanoparticles for highly sensitive electrochemical detection of chromium (VI). *Analytica Chimica Acta*.

[39] Tukur S. A., Yusof N. A., Hajian R., Hajian N. R. (2015). Linear sweep anodic stripping voltammetry: determination of Chromium (VI) using synthesized gold nanoparticles modified screen-printed electrode. *Journal of Chemical Sciences*.

[40] OMahony A. M., Scanlon M.´l D., Berduque A. (2005). Voltammetry of chromium (VI) at the liquid|liquid interface. *Electrochemistry Communications*.

[41] Svancara I., Foret P., Vytras K. (2004). A study on the determination of chromium as chromate at a carbon paste electrode modified with surfactants. *Talanta*.

[42] Miscoria S. A., Jacq C., Maeder T., Martin Negri R. (2012). Screen-printed electrodes for electroanalytical sensing, of chromium (VI) in strong acid media. *Sensors and Actuators B: Chemical*.

[43] Dom´ınguez-Renedo O., Ruiz-Espelt L., Garc´ıa-Astorgano N., Julia Arcos-Mart´ınez M. (2008). Electrochemical determination of chromium (VI) using metallic nanoparticle-modified carbon screen-printed electrodes. *Talanta*.

[44] Wyantuti S., Hartati Y. W., Firdaus M. L., Panatarani C., Tjokronegoro R. (2015). Fabrication of gold nanoparticles -modified glassy carbon electrode and its application for voltammetric detection of Cr (III). *International Journal of Scientific and Technology Research*.

[45] Boussemart M., van den Berg C. M. G., Ghaddaf M. (1992). The determination of the chromium speciation in sea water using catalytic cathodic stripping voltammetry. *Analytica Chimica Acta*.

[46] Narayana L., Suvarapu A., Somala R., Bobbala P., Hwang I., Reddy Ammireddy V. (2009). Simultaneous spectrophotometric determination of chromium (VI) and vanadium (V) by using 3,4-dihydroxybenzaldehyde isonicotinoyl hydrazone (3,4-DHBINH). *E–Journal of Chemistry*.

[47] Khan M. R., Khoo S. B. (2001). Optimization of the simultaneous batch determinations of Bi (iii), Hg (ii) and Cu (ii) at an epoxy-graphite electrode bulk modified with 2-mercaptobenzothiazole. *The Analyst*.

[48] Hutton E. A., Hočevar S. B., Ogorevc B. (2005). Ex situ preparation of bismuth film microelectrode for use in electrochemical stripping microanalysis. *Analytica Chimica Acta*.

[49] Naseri N. G., Baldock S. J., Economou A., Nicholas J., Goddard P. R. F. (2008). Disposable electrochemical flow cells for catalytic adsorptive stripping voltammetry CAdSV at a bismuth film electrode. *Analytical Bioanalytical Chemistry*.

[50] Taverniers I., De Loose M., Van Bockstaele E. (2004). Trends in quality in the analytical laboratory. II. Analytical method validation and quality assurance. *Trends in Analytical Chemistry*.

[51] Dean J. R. (2003). *Method for Environment Trace Analysis*.

