# Supplementary material for: Determination of Chromium in Natural Water by Adsorptive Stripping Voltammetry Using In Situ Bismuth Film Electrode
Source: J Environ Public Health. 2020 May 14;2020:1347836. doi: 10.1155/2020/1347836 (PMC7244981; doi:10.1155/2020/1347836)
Supplement: Supplementary Materials — Table S1: for the DP-AdSV and SqW-AdSV using in situ BiFE. Table S2: suitable experimental conditions for DP-AdSV and SqW-AdSV methods using in situ BiFE. Table S3: influence of chromium(III). Figure S1: SqW-AdSV/BiFE in situ stripping voltammograms of chromium(VI) when examining the effects of chromium(III). Table S4: influence of Fe(III) and Ca(II). Table S5: influence of Cl− and SO42−. Table S6: influence of Triton X-100. Figure S2: diagram of CCr(VI + III) analysis procedure in water sample by SqW-AdSV/in situ BiFE method. . [file 1347836.f1.pdf]

Table S1. Initially fixed experimental conditions for the DP-AdSV and SqW-AdSV using *in-situ* BiFE

N⁰	*Parameters (units of measurement)*	*Sign*	*DP -AdSV*	*SqW-AdSV*
1	Deposition potential (mV)	E_{dep}	-800	-800
2	Deposition time (s)	t_{dep}	120	120
3	Rotating rate of electrode (rpm)	ω	1500	1500
4	Equilibration time (s)	t_{equal}	30	60
5	Potential range (mV - mV)	E_{range}	-800 ÷ -1450	-800 ÷ -1450
6	Sweep rate (mV/s)	v	15	210
7	Cleaning potential (mV)	E_{clean}	300	300
8	Cleaning time (s)	t_{clean}	30	30
9	Technic parameters of stripping voltammetry<br>• Pulse amplitude (mV)<br>• Voltage step time (s)<br>• Voltage step (mV)<br>• Pulse time (ms)<br>• Frequency (Hz)	 ΔE<br>t_{step}<br>U_{step}<br>t_{pulse}<br>f	 50<br>0.4<br>6<br>40 	 30<br>-<br>6<br>-<br>35

Table S2. Suitable experimental conditions for DP-AdSV and SqW-AdSV methods using *in situ* BiFE

N⁰	*Parameters (units of measurement)*	*Sign*	*DP -AdSV*	*SqW-AdSV*
1	Deposition potential (mV)	E_{dep}	-800	-800
2	Deposition time (s)	t_{dep}	50	160
3	Rotating rate of electrode (rpm)	ω	2000	2000
4	Equilibration time (s)	t_{equal}	50	50
5	Potential range (mV - mV)	E_{range}	-800 ÷ -1450	-800 ÷ -1450
6	Sweep rate (mV/s)	v	15	210
7	Cleaning potential (mV)	E_{clean}	300	400
8	Cleaning time (s)	t_{clean}	110	100

Table S3. Influence of chromium(III)

$C_{Cr(III)}$ (ppb)	I_p (µA) (n = 2)	RE (%)
0	56,0	0
15	58,8	4,9
30	61,9	10,6
45	63,8	13, 9
60	65,7	17,4

Conditions: *$C_{Cr(VI)}$= 3.8.10^{-9}M = 0.2 ppb; $C_{Bi(III)}$= 28.8.10^{-5} M; t_{ad} = 120 s; E_{clean} = 400 mV; t_{clean} = 100 s; U_{step} = 6 mV; v = 210 mV/s; ΔE = 30 mV; f = 35 Hz.*

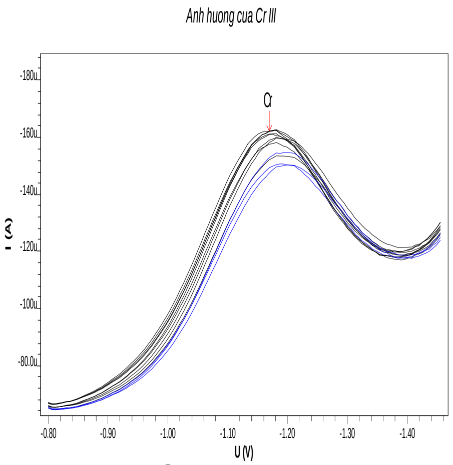

Figure S1. *SqW-AdSV/BiFE in situ stripping voltammograms of chromium (VI) when examining the effects of chromium (III)*

Table S4. Influence of Fe(III) và Ca(II)

Cation / N^0	Fe(III)			Ca(II)		
	$C_{Fe(III)}$(10^{-6} M)	$I_{p(Cr)}$ (µA)	RE (%) (n=3)	$C_{Ca(II)}$(10^{-6}M)	$I_{p(Cr)}$ (µA)	RE(%) (n=3)
1	0	66.7	0	0	103.8	0
2	9.0	67.9	1.9	12.5	103.9	0.1
3	18.0	71.8	7.6	25.0	114.3	10.1
4	27.0	70.2	5.3	37.5	115.3	11.1
5	36.0	66.3	0.6	50.0	120.9	16.4

Experimental conditions are similar to Table 1

Table S5. Influence of Cl^- và SO_4^{2-}

C_{Cl-}(10^{-3}M)	I_p (µA)	RE (%) (n =3)	C_{SO42-} (10^{-3}M)	I_p (µA)	RE (%) (n =3)
0	72.1	0	0	90.7	0
5.63	74.4	3.1	1.04	100.7	11.0
14.09	88.9	23.1	2.08	101.4	11.8
28.17	101.4	40.5	5.2	115.3	27.1
281.7	102.1	41.4	10.4	115.2	26.9

Experimental conditions are similar to Table 1

Table S6. Influence of Triton X-100

$C_{Triton\ X\text{-}100}$ (10^{-9} M)	I_p (μA)	RE (%) (n=2)
0	108,3	0
15.5	108,6	0,3
31.0	109,5	1,1
62.0	112,3	3,7
93.0	116,1	7,3

Experimental conditions are similar to Table 1

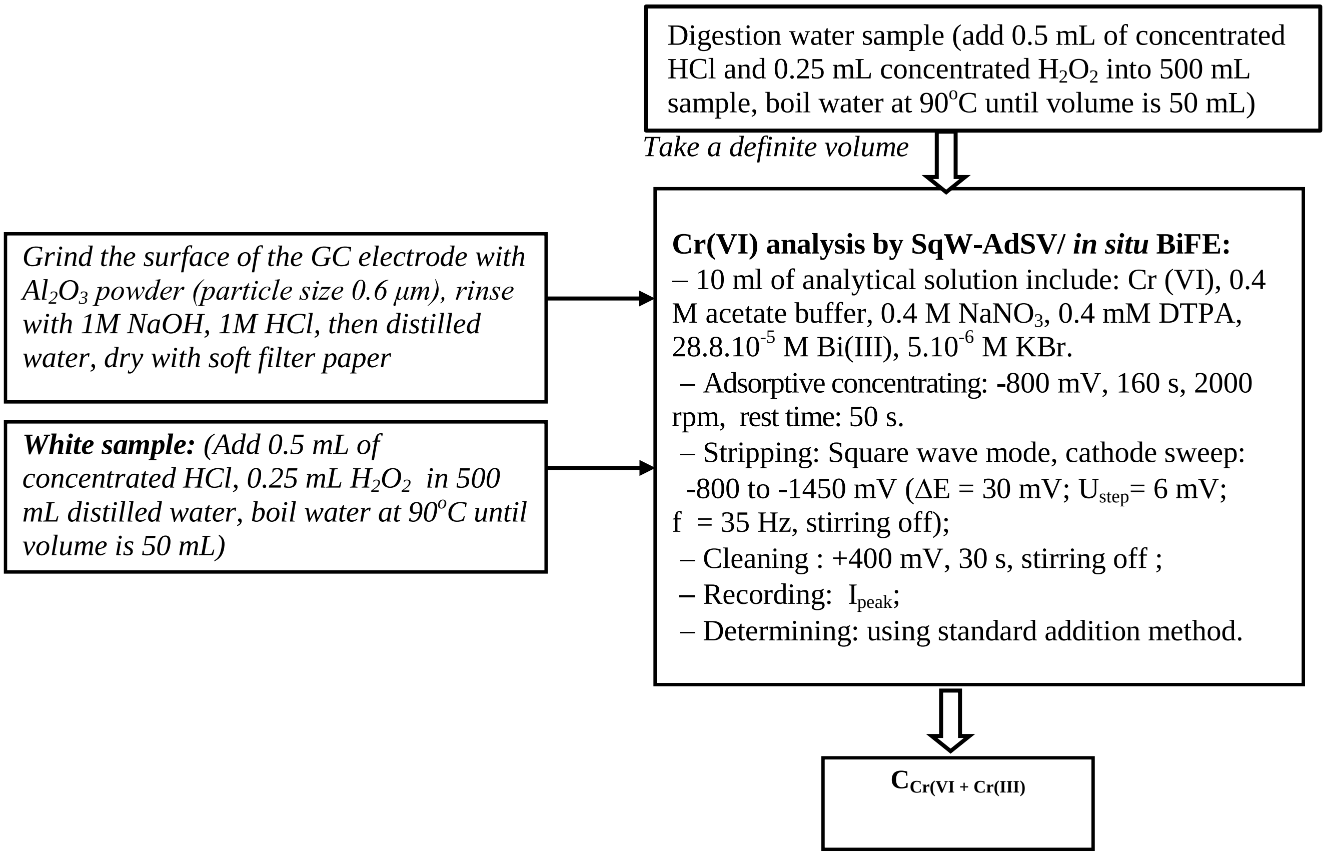

Figure S2. *Diagram of* $\mathbf{C_{Cr(VI+III)}}$ *analysis procedure in water sample by SqW-AdSV/ in situ BiFE method.*

Table S1 shows the initially fixed experimental conditions for the differential pulse adsorptive stripping voltammetry (DP-AdSV) and square wave adsorptive stripping voltammetry (SqW-AdSV) using *in-situ* BiFE. The second column is the name of the technic parameters, the fourth column is the initial value chosen for the DP-AdSV, the fifth column is the value selected for the SqW-AdSV.

Table S2 shows the suitable experimental conditions for DP-AdSV and SqW-AdSV using *in situ* BiFE after the survey using the univariate method.

Table 3 shows the influence of chromium(III) on the stripping peak current of chromium (VI) (Ip). When increasing the concentration of Cr (III) from 0 to 60 ppb, Ip does not change much under the condition of $C_{Cr(VI)}$ = 0.2 ppb; $C_{Bi(III)}$= 28.8.10^{-5} M;t_{ad} = 120 s; E_{clean} = 400 mV; t_{clean} = 100 s;U_{step} = 6 mV; v = 210 mV/s; ΔE = 30 mV; f = 35 Hz.

RE is relative error values of stripping peak current.. RE for Ip (or C) was accepted when it was equal ½ Horwitz function RSD ($RE_{Ip(Cr)} \leq ½ RSD_{Horwitz} = ½.2^{(1-0.5lgC)}$ = 32 % with C = 0.2 ppb). RE was calculated as shown in the equation 1.

$$RE_{Ip(Cr)}(\%) = \frac{[Ip(Cr) - Ip(Cr)^0]}{Ip(Cr)^0} * 100 \qquad (1)$$

$Ip(Cr)^0$ is stripping peak current without adding interferences, Ip(Cr) is stripping peak current with adding interferences.

Figure S1 is SqW-AdSV/ *in situ* BiFE stripping voltammograms of chromium (VI) when examining the effects of chromium (III). The horizontal axis represents the sweep potential from -0.8 V to - 1.45 V. The vertical axis is the intensity of the stripping peak current.

Table S4 shows the influence of Fe(III) và Ca(II) on the Ip. When increasing the concentration of Fe(III) from 0 to 36. 10^{-6} M and Ca(II) from 0 to 50.10^{-6} M, Ip does not change much under the mentioned experimental conditions. So they do not affect the determination of Cr(VI) with RE < 17%.

Table S5 shows the influence of Cl^- và SO_4^{2-} on the stripping peak current of chromium (VI). When C_{Cl-} is in the range of 0 to 14.9. 10^{-3} M, Cl^- does not affect the determination of Cr(VI). When $C_{Cl-} > 29.8$ .10^{-3} M (nearly equivalent to Cl^- concentration in brackish water), Cl^- affects the determination of Cr(VI) with RE > 32%, it is necessary to take Cl^- removal method from the sample. SO_4^{2-} did not affect the determination of Cr(VI) by SqW-AdSV / *in situ* BiFE method with RE < 27%.
